# The *Nazeris* fauna of the Nanling Mountain Range, China (Coleoptera, Staphylinidae, Paederinae)

**DOI:** 10.3897/zookeys.1059.72240

**Published:** 2021-09-08

**Authors:** Xiao-Bin Lin, Jia-Yao Hu

**Affiliations:** 1 Department of Biology, College of Life Sciences, Shanghai Normal University, 100 Guilin Road, 1st Educational Building 423-A Room, Shanghai, 200234 China Shanghai Normal University Shanghai China

**Keywords:** Flightless, leaf litter, new species, Oriental Region, rove beetles, taxonomy

## Abstract

Fourteen species of *Nazeris* Fauvel, 1873 are reported for the Nanling Mountain Range, China. Four of them are described as new: *N.xingmini* Lin & Hu, **sp. nov.** (Guangdong, Jiangxi), *N.huaiweni* Lin & Hu, **sp. nov.** (Guangdong), *N.meihuaae* Lin & Hu, **sp. nov.** (Guangdong, Jiangxi) and *N.lichongi* Lin & Hu, **sp. nov.** (Hunan). An identification key to the *Nazeris* species reported for the Nanling Mountains, and a map showing their distribution are provided.

## Introduction

The speciose paederine genus *Nazeris* Fauvel, 1873 previously included 191 species and one subspecies in China. The genus is distinguished from other paederines particularly by the morphology of the aedeagus, which has a pair of dorso-lateral apophyses (Assing 2009). All the known species of the genus are micropterous and flightless. Therefore, most of them have very narrow distributions.

The Nanling Mountain Range in central and eastern China, extend more than 500 km through Guangxi, Hunan, Guangdong and Jiangxi provinces, with several peaks of more than 1500 m. To the present, fifteen *Nazeris* species have been described from Nanling Mountain Range (Assing 2014, 2016; Hu and Li 2017; Hu et al. 2018a; Hu and Qiao 2019). During recent field trips in the Nanling Mountains, many specimens of *Nazeris* were collected. Among them, four new species were found. In the present paper, we describe the new species and provide illustrations of their major diagnostic features.

## Material and methods

The type material is deposited in the Insect Collection of the Shanghai Normal University, Shanghai, China (SNUC). The dissected body parts were mounted in Euparal on plastic slides. The habitus photos were taken using a Canon 7D camera. The photos of the sternites and aedeagi were taken using a Canon G9 camera mounted on an Olympus CX31 microscope. The original map source was obtained from http://www.simplemappr.net, an on-line tool for creating maps that can be freely used for publications and presentations.

### Measurements

Body length: measured from the anterior margin of the labrum to the apex of the abdomen

Length of forebody: measured from anterior margin of labrum to the posterior margin of the elytra

Eye length: longitudinal length of eye in dorsal view

Postocular length: measured from posterior margin of eye to posterior constriction of head in dorsal view

Head width: width of head across (and including) eyes

Head length: measured from clypeal anterior margin to posterior constriction of head

Pronotum width: maximal width of pronotum

Pronotum length: measured in midline from front margin to posterior margin

Width of elytra: combined width of elytra at posterior margin

Length of elytra: measured from apex of scutellum to posterior margin.

## Results

### Nazeris alatus

Taxon classificationAnimaliaColeopteraStaphylinidae

Hu & Li, 2017

3BD56587-23C2-5236-A687-E1A69A83ACB0

Fig. 27

#### Non-type material examined.

**China: Guangxi Prov.**: Guilin, Huaping N. R.: 5 ♂♂, 4 ♀♀, nr. Guangfu Peak, 24°33'36.57"N, 109°55'40.81"E, ca 1800 m, 22.iv.2021, sifted, Yin, Zhang, Pan and Shen leg.; 4 ♂♂, 3 ♀♀, Yunxi Valley, 25°34'00.62"N, 109°56'19.59"E, 1460–1550 m, 23.iv.2021, sifted, Yin, Zhang, Pan and Shen leg. (SNUC).

#### Comparative notes.

*Nazerisalatus* is very similar to *N.yanzhuqii* Hu & Qiao, 2019 in general appearance, but can be separated by the midline of the pronotum with short and narrow impunctate elevation posteriorly (Hu and Li 2017: 337, fig. 15); by the shorter and narrower ventral process of the aedeagus, and by the narrower dorso-lateral apophyses of the aedeagus (Hu and Li 2017: 337, fig. 18).

#### Distribution and habitat data.

The species is known only from Huaping in northeast Guangxi (Fig. 27). The specimens were collected by sifting leaf litter at altitudes of 1700–1800 m.

### Nazeris gaoleii

Taxon classificationAnimaliaColeopteraStaphylinidae

Hu, Luo & Li, 2018

21426B2E-65C3-53E1-809D-7963EF160457

Fig. 27

#### Non-type material examined.

**China: Guangdong Prov.**: Shaoguan, Nanling N. R.: 1 ♂, 1 ♀, 24°56'38"N, 112°59'31"E, 1316–1575 m, 29.vi.2020, Xia, Zhang, Yin and Lin leg.; 2 ♀♀, Guangdong Diyifeng, 24°55'29.62"N, 112°59'31.42"E, 1538–1784 m, 28.vi.2020, Xia, Zhang, Yin and Lin leg.; 1 ♂, Xiaohuangshan, 24°53'58"N, 113°01'27"E, 1,425 m, 23.viii.2020, sifted, Zhong Peng leg.; **Hunan Prov.**: Yizhang, Mangshan N. R.: 3 ♂♂, 3 ♀♀, Mengkengshi, 24°55'10"N, 112°58'37"E, 1625 m, 28.viii.2020, sifted, Zhong Peng leg.; 1 ♂, Jiangjunzhai, 24°57'03"N, 112°55'37"E, 1220 m, 27.viii.2020, sifted, Zhong Peng leg. (SNUC).

#### Comparative notes.

*Nazerisgaoleii* is very similar to *N.jiaweii* Hu, Liu & Li, 2018b in general appearance and aedeagal characters, but can be separated by the narrower posterior excision of male sternite VIII (Hu et al. 2018a: 179, fig. 21), and much shorter dorso-lateral apophyses of the aedeagus (Hu et al. 2018a: 179, fig. 22).

#### Distribution and habitat data.

The species is known from Nanling in northern Guangdong and Mangshan in southern Hunan (Fig. 27). The specimens were collected by sifting leaf litter at altitudes of 1220–1820 m.

### Nazeris huapingensis

Taxon classificationAnimaliaColeopteraStaphylinidae

Hu & Li, 2017

1F530ADD-2FBC-507F-B37D-91BA9D1A62FB

Fig. 27

#### Non-type material examined.

**China: Guangxi Prov.**: Guilin, Huaping N. R.: 6 ♂♂, 9 ♀♀, nr. Guangfu Peak, 24°33'36.57"N, 109°55'40.81"E, ca 1800 m, 22.iv.2021, sifted, Yin, Zhang, Pan and Shen leg.; 8 ♂♂, 11 ♀♀, Yunxi Valley, 25°34'00.62"N, 109°56'19.59"E, 1460–1550 m, 23.iv.2021, sifted, Yin, Zhang, Pan and Shen leg. (SNUC).

#### Comparative notes.

*Nazerishuapingensis* is very similar to *N.obtortus* Assing, 2016 from the same locality in general appearance and separated only by aedeagal characters: the apex of the ventral process in ventral view is much broader; apices of the dorso-lateral apophyses rounder and broader (Hu and Li 2017: 336, fig. 13).

#### Distribution and habitat data.

The species is known only from Huaping in northeast Guangxi (Fig. 27). The specimens were collected by sifting leaf litter at altitudes of 1300–1800 m.

### Nazeris latilobatus

Taxon classificationAnimaliaColeopteraStaphylinidae

Assing, 2016

0B669BA2-CA6F-5D21-961F-4A04B5496CDC

Fig. 27

#### Non-type material examined.

**China: Guangxi**: Xing’an, Mao’ershan N. R.: 1 ♂, 25°52'29.52"N, 110°28'20.01"E, 528 m, 25.viii.2020, Chong Li leg.; 2 ♂♂, 25°30'15.72"N, 110°25'50.87"E, 1900–2040 m, 27.viii.2020, Lu Qiu leg.; 20 ♂♂, 16 ♀♀, Antangping, 25°54'44.07"N, 110°27'37.68"E, 1660 m, 6–7.v.2021, sifted, Yin, Zhang, Pan and Shen leg.; 1 ♂, botanical garden, 25°53'03.83"N, 110°29'13.53"E, 1160 m, 8.v.2021, sifted, Yin, Zhang, Pan and Shen leg. (SNUC).

#### Comparative notes.

*Nazerislatilobatus* is similar to *N.qini* Hu & Li, 2012 from Dayaoshan in external and the male sexual characters, but can be separated by the deeper posterior excision of the male sternite VIII (Assing 2016: 307, fig. 9), and the much broader apex of the aedeagal ventral process (Assing 2016: 307, fig. 11).

#### Distribution and habitat data.

The species is known only from Mao’ershan in northeast Guangxi (Fig. 27). The specimen was collected by sifting leaf litter at altitudes of 450–2040 m.

### Nazeris nanlingensis

Taxon classificationAnimaliaColeopteraStaphylinidae

Hu, Luo & Li, 2018

45448EF1-28A2-518A-8ED5-C6E0AC6BA9AE

Fig. 27

#### Non-type material examined.

**China: Guangdong Prov.**: Shaoguan, Nanling N. R.: 2 ♂♂, 1 ♀, 24°56'38"N, 112°59'31"E, 1316–1575 m, 29.vi.2020, Xia, Zhang, Yin and Lin leg.; 2 ♂♂, 2 ♀♀, Xiaohuangshan, 24°53'58"N, 113°01'27"E, 1425 m, 23.viii.2020, sifted, Zhong Peng leg.; **Hunan Prov.**: Yizhang County, Mangshan N. R.: 2 ♂♂, 1 ♀, Mengkengshi, 24°55'10"N, 112°58'37"E, 1625 m, 28.viii.2020, sifted, Zhong Peng leg.; 6 ♂♂, 5 ♀♀, Jiangjunzhai, 24°57'03"N, 112°55'37"E, 1220 m, 27.viii.2020, sifted, Zhong Peng leg. (SNUC).

#### Comparative notes.

*Nazerisnanlingensis* is very similar to *N.rubidus* Hu, Luo & Li, 2018a from the same locality in general appearance and aedeagal characters, but can be separated by the smaller forebody size; less dense punctation of the head and pronotum (Hu et al. 2018a: 177, fig. 14); a wider ventral process and slenderer dorso-lateral apophyses of the aedeagus in ventral view (Hu et al. 2018a: 177, fig. 17).

#### Distribution and habitat data.

The species is known from Nanling in northern Guangdong and Mangshan in southern Hunan (Fig. 27). The specimens were collected by sifting leaf litter at altitudes of 1100–1850 m.

### Nazeris obtortus

Taxon classificationAnimaliaColeopteraStaphylinidae

Assing, 2016

4EE6888F-ACC4-549A-8174-797BAAF6AF56

Fig. 27

#### Non-type material examined.

**China: Guangxi Prov.**: Guilin, Huaping N. R.: 1 ♂, 3 ♀♀, Hongtan, 25°36'15"N, 109°57'35"E, 820–950 m, 24.iv.2021, sifted, Yin, Zhang, Pan and Shen leg. (SNUC).

#### Comparative notes.

*Nazerisobtortus* is quite similar to *N.huapingensis* and separated only by aedeagal characters: the apex of the ventral process and apices of the dorso-lateral apophyses are much narrower (Assing 2016: 309, fig. 16).

#### Distribution and habitat data.

The species is known only from Huaping in northeast Guangxi (Fig. 27). The specimens were collected by sifting leaf litter at altitudes of 820–1200 m.

### Nazeris rubidus

Taxon classificationAnimaliaColeopteraStaphylinidae

Hu, Luo & Li, 2018

0C0FB2F9-5E54-5039-864D-9F4CB6EBBCC1

Fig. 27

#### Non-type material examined.

**China: Guangdong Prov.**: Shaoguan, Nanling N. R.: 5 ♂♂, 7 ♀♀, 24°56'38"N, 112°59'31"E, 1316–1575 m, 29.vi.2020, Xia, Zhang, Yin and Lin leg.; ♂♂, 43 ♀♀, 24°55'43.67"N, 113°0'58.50"E, 1,020 m, 27.vi.2020, Xia, Zhang, Yin and Lin leg.; 2 ♂♂, 5 ♀♀, Xiaohuangshan, 24°53'58"N, 113°01'27"E, 1425 m, 23.viii.2020, sifted, Zhong Peng leg. (SNUC).

#### Comparative notes.

*Nazerisrubidus* is very similar to *N.huapingensis* in general appearance and aedeagal characters, but can be separated by the following combination of characters: the posterior excision of the male sternite VIII is wider (Hu et al. 2018a: 176, fig. 11); the apex of the ventral process of the aedeagus is widely rounded in ventral view (Hu et al. 2018a: 176, fig. 12); the dorso-lateral apophyses is nearly straight in lateral view (Hu et al. 2018a: 176, fig. 13).

#### Distribution and habitat data.

The species is known from Nanling in northern Guangdong and Mangshan in southern Hunan (Fig. 27). The specimens were collected by sifted leaf litter at altitudes of 700–1820 m.

### Nazeris rugosus

Taxon classificationAnimaliaColeopteraStaphylinidae

Hu & Qiao, 2019

F98DCD01-4E91-5FE1-9675-2982DB8FEB15

Fig. 27

#### Non-type material examined.

**China: Guangxi Prov.**: Xing’an, Mao’ershan N. R.: 1 ♂, 25°51'57.56"N, 110°24'46.19"E, 2100 m, 5.v.2021, bamboo, broad-leaved bush, sifted, Yin, Zhang, Pan and Shen leg.; 1 ♂, Lijiangyuan, 25°53'32.64"N, 110°25'41.68"E, 1990–2030 m, 6.v.2021, sifted, Yin, Zhang, Pan and Shen leg. (SNUC).

#### Comparative notes.

*Nazerisrugosus* is distinguished from all the known species of *Nazeris* from the Nanling Mountains by the microsculpture covering the head, pronotum and abdomen (Hu and Qiao 2019: 436, figs 18–20), and by the distinctive shape of the aedeagus, particularly the apically narrowed ventral process (Hu and Qiao 2019: 436, fig. 23).

#### Distribution and habitat data.

The species is known only from Mao’ershan in northeast Guangxi (Fig. 27). The specimen was collected by sifting leaf litter at altitudes of 1990–2100 m.

### Nazeris yanzhuqii

Taxon classificationAnimaliaColeopteraStaphylinidae

Hu & Qiao, 2019

9D948F4A-5BB9-5714-9CAB-471F3E52A7B1

Fig. 27

#### Non-type material examined.

**China: Guangxi Prov.**: Xing’an, Mao’ershan N. R.: 1 ♀, Lijiangyuan, 25°53'32.64"N, 110°25'41.68"E, 1990–2030 m, 6.v.2021, sifted, Yin, Zhang, Pan and Shen leg. (SNUC).

#### Comparative notes.

*Nazerisyanzhuqii* is most similar to *N.alatus* in general appearance and aedeagal characters, but can be separated by the impunctate elevation of the pronotum very narrow or absent (Hu and Qiao 2019: 438, fig. 35), by the longer and wider ventral process of the aedeagus in ventral view, with much smaller basal laminae, and by the wider dorso-lateral apophyses of the aedeagus (Hu and Qiao 2019: 438, fig. 38).

#### Distribution and habitat data.

The species is known only from Mao’ershan in northeast Guangxi (Fig. 27). The specimen was collected by sifting leaf litter at altitudes of 1940–2140 m.

### Nazeris yuyimingi

Taxon classificationAnimaliaColeopteraStaphylinidae

Hu & Qiao, 2019

38F5A8F6-874A-5431-8CE2-364F665A840D

Fig. 27

#### Non-type material examined.

**China: Guangxi Prov.**: Xing’an, Mao’ershan N. R.: 1 ♀, nr. Antangping, 25°54'44.07"N, 110°27'37.68"E, 1660 m, 7.v.2021, sifted, Yin, Zhang, Pan and Shen leg. (SNUC).

#### Comparative notes.

*Nazerisyuyimingi* is similar in general appearance and aedeagal characters to *N.chenyanae* Hu & Li, 2017, but can be separated by the shallowly emarginate male sternite VII (Hu and Qiao 2019: 437, fig. 26), by the narrower ventral process and the wider apex of the dorso-lateral apophyses of the aedeagus in ventral view (Hu and Qiao 2019: 437, fig. 28).

#### Distribution and habitat data.

The species is known only from Mao’ershan in northeast Guangxi (Fig. 27). The specimen was collected by sifting leaf litter at altitudes of 1143–1660 m.

### Nazeris xingmini

Taxon classificationAnimaliaColeopteraStaphylinidae

Lin & Hu sp. nov.

E21EDC2E-DFC2-5E96-B6C1-D5ACC9AE7473

http://zoobank.org/C2BCCA84-E6E7-430C-BEE6-D7A6FA183751

Figs 1 , 5–9 , 27

#### Type material.

***Holotype*: China**: ♂: “China: Guangdong Prov., Shixing County, Chebaling N. R., 24°43'22"N, 114°15'22"E, 357 m, 19.viii.2020, Liang Tang leg.” (SNUC). ***Paratypes***: 1 ♂, same data as holotype; 1 ♂, 2 ♀♀, “China: Jiangxi Prov., Longnan County, Jiulianshan N. R., 24°30'59.23"N, 114°24'52.98"E, alt. 587 m, 16.viii.2020, Liang Tang leg.” (SNUC).

**Figures 1–4.**
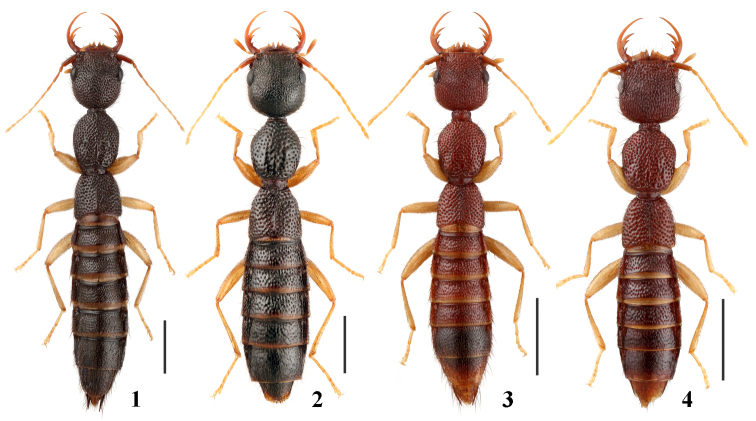
Male habitus of *Nazeris* spp **1***N.xingmini***2***N.huaiweni***3***N.meihuaae***4***N.lichongi*. Scale bars: 1.0 mm.

#### Description.

Body length 6.4–7.5 mm; forebody length 3.2–3.4 mm.

Body (Fig. 1) dark brown; legs yellowish brown; antennae dark brown to light brown.

**Figures 5–9.**
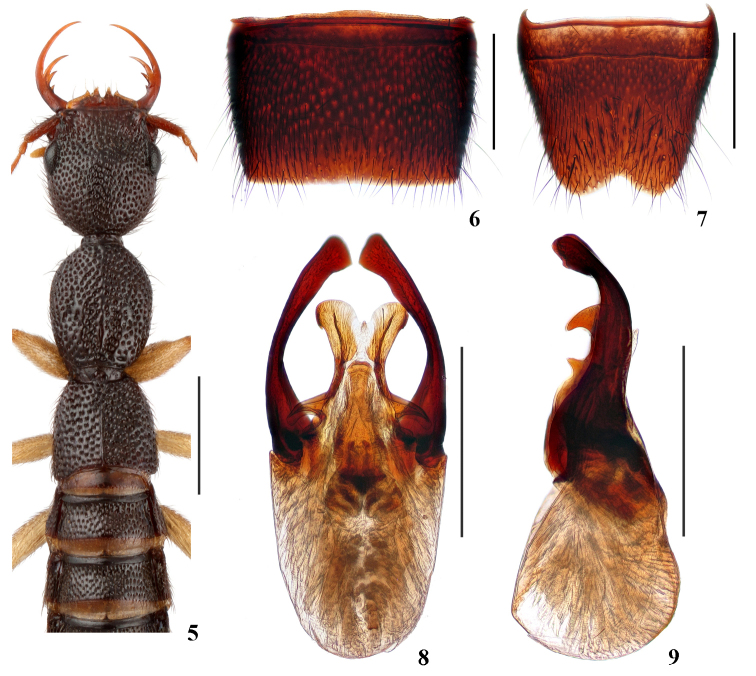
*Nazerisxingmini***5** forebody **6** male sternite VII **7** male sternite VIII **8** aedeagus in ventral view **9** aedeagus in lateral view. Scale bars: 1.0 mm (**5**); 0.5 mm (**6–9**).

Head (Fig. 5) 1.02–1.12 times as long as wide; punctation very dense, moderately coarse, non-umbilicate, interstices lacking microsculpture; postocular portion approximately 1.6–2.1 times as long as eye length.

Pronotum (Fig. 5) 1.18–1.22 times as long as wide, 0.95–1.02 times as long and 0.83–0.91 times as broad as head; punctation non-umbilicate, moderately dense and as coarse as that of head; midline posteriorly with short and very narrow impunctate elevation; interstices lacking microsculpture.

Elytra (Fig. 5) 0.59–0.67 times as long as wide, 0.50–0.57 times as long and 0.96–1.09 times as broad as pronotum; punctation as dense as, and slightly coarser than that of pronotum; interstices lacking microsculpture.

Abdomen with punctation dense and rather coarse on tergites III–V, dense and less coarse on tergite VI, moderately dense and fine on tergites VII–VIII; interstices lacking microsculpture.

**Male.** Sternite VII (Fig. 6) with posterior margin truncate at middle. Sternite VIII (Fig. 7) with wide triangular posterior excision. Aedeagus (Figs 8, 9) well sclerotized; with ventral process narrowed near middle in ventral view, with U-shaped excision at apex in ventral view, with pair of wing-like basal laminae ventrally; dorso-lateral apophyses moderately strong, distinctly curved in ventral view, curved dorsally and slightly widened at apices in lateral view, extending beyond apex of ventral process.

#### Distribution and habitat data.

The species is known from Chebaling in northern Guangdong and Jiulianshan in southern Jiangxi (Fig. 27). The specimens were collected by sifting leaf litter at altitudes of 357–587 m.

#### Comparative notes.

The new species is very similar to *N.inaequalis* Assing, 2014 in general appearance and separated only by the aedeagal characters: the apex of the ventral process is symmetric in ventral view (Fig. 8); dorso-lateral apophyses extending beyond the apex of the ventral process.

#### Etymology.

The species is named in honor of Xing-Min Wang (South China Agricultural University) who helped a lot during our collection in Nanling.

### Nazeris huaiweni

Taxon classificationAnimaliaColeopteraStaphylinidae

Lin & Hu sp. nov.

5A5DC91C-A3F3-5C87-9501-0B8F9D6C93A0

http://zoobank.org/2C276CF2-9719-40ED-A290-B50052066D15

Figs 2 , 10–14 , 27

#### Type material.

***Holotype*: China**: ♂: “China: Guangdong, Shaoguan, Nanling N. R., 24°56'38"N, 112°59'31"E, 1316–1575 m, 29.vi.2020, Xia, Zhang, Yin and Lin leg.” (SNUC). ***Paratypes***: 2 ♀♀, same data, except “Ruyuan, Nanling, nr. Ruyang, Xiaohuangshan, 24°53'44.7"N, 113°1'26.9"E, 1270–1570 m, 2021.v.02, Hu, Lin, Zhou and Li leg.” (SNUC).

#### Description.

Body length 6.2–6.8 mm; forebody length 3.2–3.3 mm.

Body (Fig. 2) dark brown; antennae and legs yellowish brown.

**Figures 10–14.**
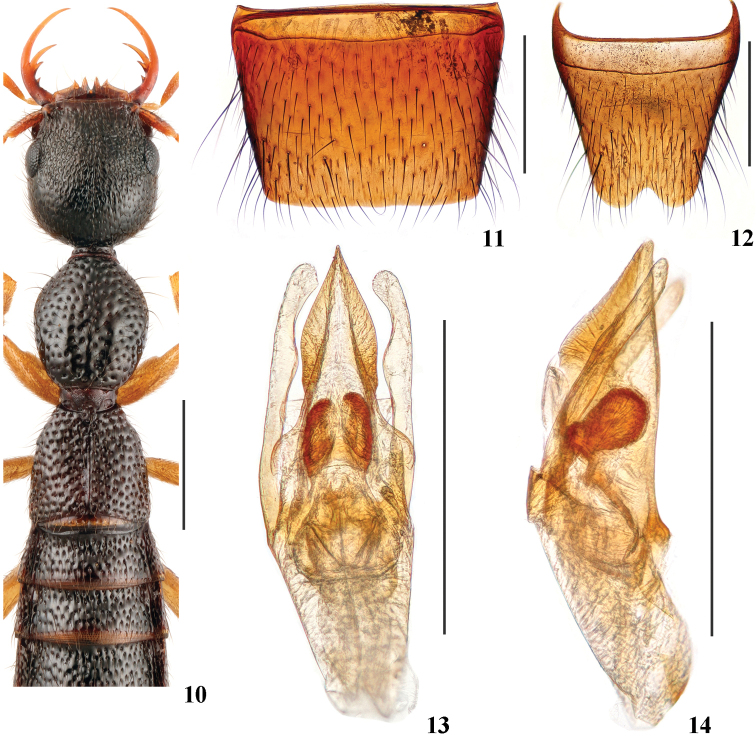
*Nazerishuaiweni***10** forebody **11** male sternite VII **12** male sternite VIII **13** aedeagus in ventral view **14** aedeagus in lateral view. Scale bars: 1.0 mm (**10**); 0.5 mm (**11–14**).

Head (Fig. 10) approximately as long as wide; punctation very dense, moderately coarse, distinctly umbilicate, interstices lacking microsculpture; postocular portion approximately twice as long as eye length.

Pronotum (Fig. 10) 1.12–1.21 times as long as wide, 0.93–1.02 times as long and 0.83–0.84 times as broad as head; punctation non-umbilicate, moderately dense and as coarse as that of head; midline posteriorly with short and very narrow impunctate elevation; interstices lacking microsculpture.

Elytra (Fig. 10) 0.67–0.76 times as long as wide, 0.69–0.71 times as long and 1.03–1.12 times as broad as pronotum; punctation slightly denser and coarser than that of pronotum; interstices lacking microsculpture.

Abdomen with punctation dense and rather coarse on tergites III–V, dense and less coarse on tergite VI, moderately dense and fine on tergites VII–VIII; interstices lacking microsculpture.

**Male.** Sternite VII (Fig. 11) with posterior margin truncate at middle. Sternite VIII (Fig. 12) with triangular posterior excision. Aedeagus (Figs 13, 14) with ventral process gradually narrowed in apicad half, with acute apex in ventral view or lateral view, with pair of wing-like basal laminae ventrally; dorso-lateral apophyses moderately slender, slightly widened near middle and apices in ventral view, not reaching apex of ventral process.

#### Distribution and habitat data.

The species is known only from Nanling in northern Guangdong (Fig. 27). The specimens were collected by sifted leaf litter at altitudes of 1270–1575 m.

#### Comparative notes.

The new species is very similar to *N.divisus* Hu & Li, 2015 in general appearance, but can be separated by the wider and shallower posterior excision of male sternite VIII (Fig. 12), by the ventral process with acute apex (Fig. 13), and by the wider dorso-lateral apophyses of aedeagus (Fig. 13).

#### Etymology.

The species is named in honor of Huai-Wen Wang (Administration of Nanling National Nature Reserve) who helped a lot during our collection in Nanling.

### Nazeris meihuaae

Taxon classificationAnimaliaColeopteraStaphylinidae

Lin & Hu sp. nov.

535E8780-656F-51A6-8666-65DE5686F046

http://zoobank.org/B62BB393-C4B6-41EE-96A7-2B3E49F0C50A

Figs 3 , 15–21 , 27

#### Type material.

***Holotype*: China**: ♂: “China: Guangdong Prov., Shixing County, Chebaling N. R., 24°40'41.82"N, 114°10'20.42"E, 1067 m, 20.viii.2020, Liang Tang leg.” (SNUC). ***Paratypes***: 5 ♂♂, 18 ♀♀, same data as holotype; 3 ♂♂, 8 ♀♀, same data, except “872 m, 20.viii.2020”; 2 ♀♀, “China: Guangdong Prov., Shixing County, Chebaling N. R., 24°40'58"N, 114°10'14"E, 468–870 m, 24.vi.2020, Xia, Zhang, Yin and Lin leg.”; 6 ♂♂, 3 ♀♀, “China: Jiangxi Prov., Longnan County, Jiulianshan N. R., 24°30'10"N, 114°26'35"E, 795 m, 18.viii.2020, Liang Tang leg.”; 1 ♂, 2 ♀♀, “China: Jiangxi Prov., Longnan County, Jiulianshan N. R., 24°30'10.43"N, 114°26'35.28"E, leaf litter, sifted, 1253 m, 17.viii.2020, Liang Tang leg.”; 5 ♂♂, 1 ♀, “China: Jiangxi Prov., Longnan County, Jiulianshan, Huangniushi, 24°31'22.7"N, 114°25'3.6"E, 600–1000 m, 10.v.2021, C.-L. Zhou & C. Li leg.”; 3 ♀♀, “China: Jiangxi Prov., Longnan County, Jiulianshan, summit of Huangniushi, 24°30'53"N, 114°26'6.72"E, 1000–1230 m, 12.v.2021, Zhou and Li leg.”; 1 ♂, “China: Jiangxi Prov., Longnan County, Jiulianshan, summit of Huangniushi, 24°30'53"N, 114°26'6.72"E, 1,000–1,230 m, 12.v.2021, Zhou and Li leg.” (SNUC).

#### Description.

Body length 4.1–4.8 mm; forebody length 2.2–2.6 mm.

Body (Fig. 3) reddish brown; antennae and legs yellowish brown.

**Figures 15–21.**
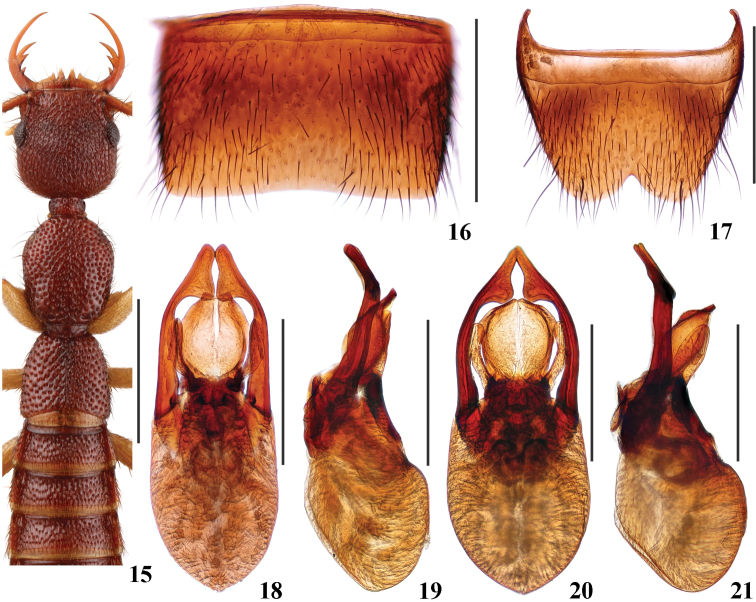
*Nazerismeihuaae* (**15–19** specimen from Chebaling **20–21** specimen from Jiulianshan) **15** forebody **16** male sternite VII **17** male sternite VIII **18, 20** aedeagus in ventral view **19, 21** aedeagus in lateral view. Scale bars: 1.0 mm (**15**); 0.5 mm (**16–21**).

Head (Fig. 15) 0.97–1.03 times as long as wide; punctation very dense, moderately coarse, distinctly umbilicate and partly confluent, interstices lacking microsculpture; postocular portion approximately 1.5–2.1 times as long as eye length.

Pronotum (Fig. 15) 1.05–1.23 times as long as wide, 0.91–1.10 times as long and 0.83–0.87 times as broad as head; punctation non-umbilicate, moderately dense and as coarse as that of head; midline posteriorly with short and very narrow impunctate elevation; interstices lacking microsculpture.

Elytra (Fig. 15) 0.61–0.75 times as long as wide, 0.54–0.66 times as long and 0.97–1.10 times as broad as pronotum; punctation as dense as, and slightly coarser than that of pronotum; interstices lacking microsculpture.

Abdomen with punctation dense and rather coarse on tergites III–V, dense and less coarse on tergite VI, moderately dense and fine on tergites VII–VIII; interstices lacking microsculpture.

**Male.** Sternite VII (Fig. 16) with posterior margin shallowly emarginate in the middle. Sternite VIII (Fig. 17) with wide triangular posterior excision. Aedeagus (Figs 18–21) with ventral process short, widened near middle in ventral view, with pair of finger-like basal laminae ventrally; dorso-lateral apophyses distinctly curved and widened in apical third in ventral view, extending beyond apex of ventral process.

#### Distribution and habitat data.

The species is known from Chebaling in northern Guangdong and Jiulianshan in southern Jiangxi (Fig. 27). The specimens were collected by sifting leaf litter at altitudes of 468–1253 m.

#### Comparative notes.

This species is very similar in general appearance and aedeagal characters to *N.pengzhongi* Hu & Li, 2015, but can be separated by the finger-like basal laminae of the ventral process and the longer dorso-lateral apophyses of the aedeagus (Figs 18, 20). The new species is also similar in general appearance to *N.rubidus* and *N.nanlingensis*, but can be separated by the distinctly longer laminae of the ventral process and the apically wider dorso-lateral apophyses of the aedeagus (Figs 18, 20). Compared with the holotype from Chebaling, Guangdong (Figs 18, 19), the specimens from Jiulianshan, Jiangxi (Figs 20, 21) display a slightly shorter ventral process and slightly narrower apices of the dorso-lateral apophyses of the aedeagus. Based on the similar general appearance and male sternites, these aedeagal differences are treated as intraspecific variation.

#### Etymology.

The species is named in honor of Mei-Hua Xia, who collected some of the type specimens.

### Nazeris lichongi

Taxon classificationAnimaliaColeopteraStaphylinidae

Lin & Hu sp. nov.

8DFD645D-9C43-5D66-941B-94D0CFDC29B8

http://zoobank.org/8DFC4E1C-104B-47C5-9511-5CB6B1C0662D

Figs 4 , 22–26 , 27

#### Type material.

***Holotype*: China**: ♂: “China: Hunan Prov., Yongzhou County, Dupangling N. R., 25°26'12.45"N, 111°20'23.29"E, 448 m, 29.viii.2020, sifted, Chong Li leg.” (SNUC).

#### Description.

Body length 4.7 mm; forebody length 2.4 mm.

Body (Fig. 4) reddish brown; antennae and legs yellowish brown.

**Figures 22–26.**
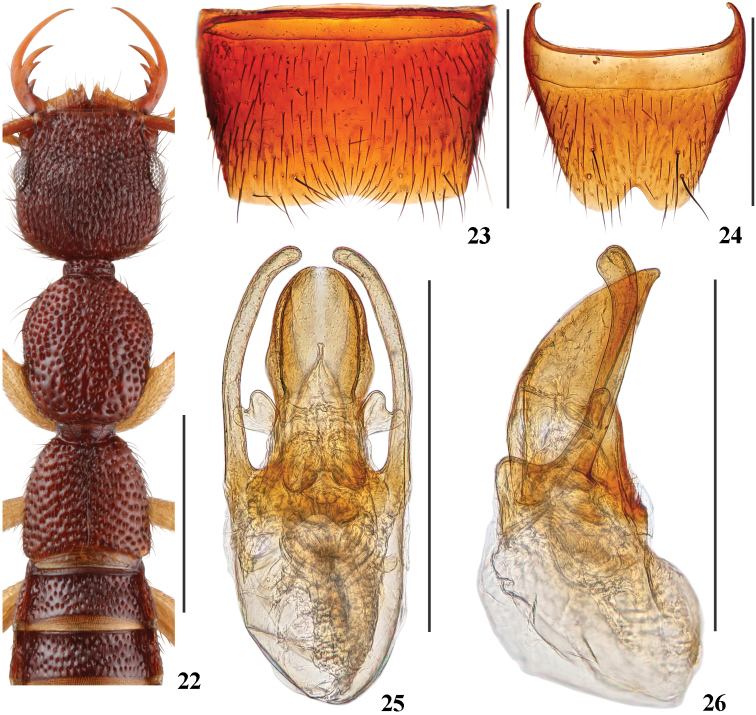
*Nazerislichongi***22** forebody **23** male sternite VII **24** male sternite VIII **25** aedeagus in ventral view **26** aedeagus in lateral view. Scale bars: 1.0 mm (**22**); 0.5 mm (**23–26**).

Head (Fig. 22) 0.97 times as long as wide; punctation very dense, moderately coarse, distinctly umbilicate and partly confluent, interstices lacking microsculpture; postocular portion approximately 1.6 times as long as eye length.

Pronotum (Fig. 22) 1.17 times as long as wide, as long as and 0.83 times as broad as head; punctation non-umbilicate, moderately dense and as coarse as that of head; midline posteriorly with short and very narrow impunctate elevation; interstices lacking microsculpture.

Elytra (Fig. 22) 0.77 times as long as wide, 0.66 times as long and as broad as pronotum; punctation as dense as, and slightly coarser than that of pronotum; interstices lacking microsculpture.

Abdomen with punctation dense and rather coarse on tergites III–V, dense and less coarse on tergite VI, moderately dense and fine on tergites VII–VIII; interstices lacking microsculpture.

**Male.** Sternite VII (Fig. 23) with posterior margin shallowly emarginate in the middle. Sternite VIII (Fig. 24) with triangular posterior excision. Aedeagus (Figs 25, 26) with broad ventral process, slightly widened in apical half, with round apex in ventral view, with pair of heart-like basal laminae; dorso-lateral apophyses slender, distinctly curved in ventral view, curved dorsally and slightly widened at apices in lateral view, extending beyond apex of ventral process.

#### Distribution and habitat data.

**Figure 27.**
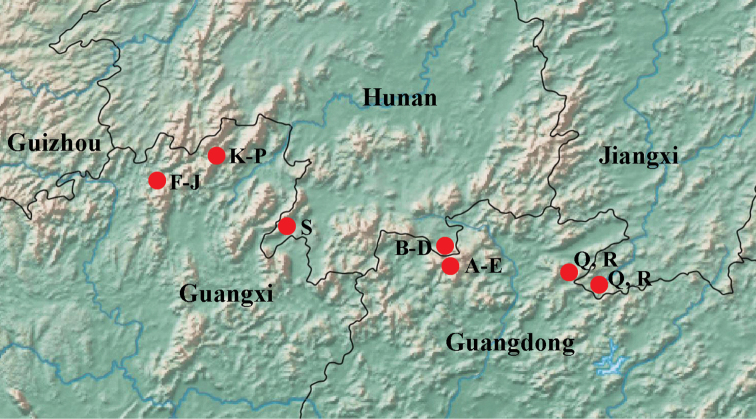
Map showing the distribution of *Nazeris* in Nanling Mountain Range **A***N.inaequalis***B***N.rubidus***C***N.nanlingensis***D***N.gaoleii***E***N.huaiweni***F***N.obtortus***G***N.huapingensis***H***N.alatus***I***N.exilis***J***N.chenyanae***K***N.latilobatus***L***N.maoershanus***M***N.rugosus***N***N.yuyimingi***O***N.biacuminatus***P***N.yanzhuqii***Q***N.xingmini***R***N.meihuaae***S***N.lichongi*.

The species is known only from Dupangling in southern Hunan (Fig. 27). The specimen was collected by sifting leaf litter at an altitude of 448 m.

The new species is similar in general appearance and aedeagal characters to *N.rubidus* and *N.nanlingensis*, but can be separated by the slightly dorsally curved dorso-lateral apophyses of the aedeagus in lateral view (Fig. 26), and by the heart-like basal laminae of the ventral process (Fig. 25).

#### Etymology.

The species is named in honor of Chong Li, who collected some of the type specimens.

### Key to *Nazeris* species in Nanling mountain range

**Table:** 

1	Head with non-umbilicate punctation (Fig. 5)	**2**
–	Head with umbilicate punctation (Fig. 15)	**6**
2	Body reddish brown, abdomen with fine microsculpture on all tergites	***N.gaoleii* Hu Luo & Li, 2018**
–	Body dark brown, abdomen lacking microsculpture	**3**
3	Pronotum with inconspicuous or lacking impunctate elevation in posterior half (Hu and Qiao 2019: 440, fig. 35); forebody length at most 2.9 mm	***N.yanzhuqii* Hu & Qiao, 2019**
–	Pronotum with narrow impunctate elevation in posterior half (Fig. 5); forebody length at least 3.1 mm	**4**
4	Ventral process of the aedeagus distinctly asymmetrical, dorso-lateral apophyses not reaching apex of ventral process (Assing, 2014: 26, fig. 58)	***N.inaequalis* Assing, 2014**
–	Ventral process of the aedeagus symmetrical, dorso-lateral apophyses extending beyond apex of ventral process	**5**
5	Dorso-lateral apophyses of aedeagus moderately strong, with widened apex (Figs 8, 9)	***N.xingmini* sp. nov.**
–	Dorso-lateral apophyses of aedeagus slender, with acute apex (Hu and Li 2017: 337, figs 18, 19)	***N.alatus* Hu & Li, 2017**
6	Body dark brown (Figs 1, 2), body length at least 6.1 mm, forebody length at least 3.2 mm	**7**
–	Body reddish brown (Figs 3, 4), body length at most 6.0 mm, forebody length at most 3.0 mm	**11**
7	Apex of ventral process of aedeagus divided into two branches in ventral view (Hu and Li 2017: 338, fig. 23)	**8**
–	Apex of ventral process of aedeagus not divided into two branches in ventral view	**9**
8	Sternite VII with posterior margin weakly protruding at middle (Hu and Li 2017: 338, fig. 21); ventral process of aedeagus with thin apical branches (Hu and Li 2017: 338, figs 23, 24); dorso-lateral apophyses of aedeagus slightly curved in lateral view (Hu and Li 2017: 338, fig. 24)	***N.exilis* Hu & Li, 2017**
–	Sternite VII with posterior margin truncate at middle (Hu and Qiao 2019: 438, fig. 31); ventral process of aedeagus with wide apical branches (Hu and Qiao 2019: 438, figs 33, 34); dorso-lateral apophyses of aedeagus straight in lateral view (Hu and Qiao 2019: 438, fig. 34)	***N.biacuminatus* Hu & Qiao, 2019**
9	Sternite VIII with rounded triangular posterior excision (Fig. 12); dorso-lateral apophyses of aedeagus not reaching apex of ventral process (Fig. 13)	***N.huaiweni* sp. nov.**
–	Sternite VIII with sharp, V-shaped posterior excision (Hu and Qiao 2019: 437, fig. 27); dorso-lateral apophyses of aedeagus extending slightly beyond apex of ventral process (Hu and Qiao 2019: 437, fig. 28)	**10**
10	Male sternite VII shallowly emarginate in the middle (Hu and Qiao 2019: 437, fig. 26); dorso-lateral apophyses of aedeagus widened near apex in ventral view (Hu and Qiao 2019: 437, fig. 28)	***N.yuyimingi* Hu & Qiao, 2019**
–	Male sternite VII not emarginate in the middle (Hu and Li 2017: 340, fig. 26); dorso-lateral apophyses of aedeagus not widened near apices in ventral view (Hu and Li 2017: 340, fig. 28)	***N.chenyanae* Hu & Li, 2017**
11	Head and pronotum with fine microsculpture (Hu and Qiao 2019: 436, figs 18, 19)	***N.rugosus* Hu & Qiao, 2019**
–	Head and pronotum lacking microsculpture	**12**
12	Dorso-lateral apophyses of aedeagus extending to same level as apex of ventral process (Hu and Qiao 2019: 434, fig. 10)	***N.latilobatus* Assing, 2016**
–	Dorso-lateral apophyses of aedeagus extending distinctly beyond apex of ventral process	**13**
13	Ventral process of aedeagus nearly triangular, with narrow apex in ventral view (Assing 2016: 309, fig. 16)	***N.obtortus* Assing, 2016**
–	Ventral process of aedeagus broad, with wide apex in ventral view	**14**
14	Basal laminae of ventral process of aedeagus very long, more than half length of ventral process (Figs 18, 20)	***N.meihuaae* sp. nov.**
–	Basal laminae of ventral process of aedeagus very short, much less than half length of ventral process	**15**
15	Ventral process of aedeagus with round apex in ventral view	**16**
–	Ventral process of aedeagus with truncate or emarginate apex in ventral view	**17**
16	Dorso-lateral apophyses of aedeagus curved ventrally in lateral view (Hu et al. 2018a: 176, fig. 13); ventral process with wing-like basal laminae (Hu et al. 2018a: 176, fig. 12)	***N.rubidus* Hu, Luo & Li, 2018**
–	Dorso-lateral apophyses of aedeagus curved dorsally in lateral view (Fig. 26); ventral process with heart-like basal laminae (Fig. 25)	***N.lichongi* sp. nov.**
17	Ventral process of aedeagus in ventral view distinctly widened in apical half (Hu et al. 2018a: 177, fig. 17)	***N.nanlingensis* Hu, Luo & Li, 2018**
–	Ventral process of aedeagus in ventral view narrowed in apical half (Hu and Li 2017: 336, fig. 13)	**18**
18	Apex of ventral process of aedeagus nearly truncate in ventral view (Hu and Li 2017: 336, fig. 13); apices of dorso-lateral apophyses roundly widened in ventral view (Hu and Li 2017: 336, fig. 13)	***N.huapingensis* Hu & Li, 2017**
–	Apex of ventral process of aedeagus with small semi-circular emargination in ventral view (Hu and Qiao 2019: 435, fig. 15); apices of dorso-lateral apophyses not widened in ventral view (Hu and Qiao 2019: 435, fig. 15)	***N.maoershanus* Hu & Qiao, 2019**

## Supplementary Material

XML Treatment for Nazeris alatus

XML Treatment for Nazeris gaoleii

XML Treatment for Nazeris huapingensis

XML Treatment for Nazeris latilobatus

XML Treatment for Nazeris nanlingensis

XML Treatment for Nazeris obtortus

XML Treatment for Nazeris rubidus

XML Treatment for Nazeris rugosus

XML Treatment for Nazeris yanzhuqii

XML Treatment for Nazeris yuyimingi

XML Treatment for Nazeris xingmini

XML Treatment for Nazeris huaiweni

XML Treatment for Nazeris meihuaae

XML Treatment for Nazeris lichongi
